# Implications of the Orb2 Amyloid Structure in Huntington’s Disease

**DOI:** 10.3390/ijms21186910

**Published:** 2020-09-21

**Authors:** Rubén Hervás, Alexey G. Murzin, Kausik Si

**Affiliations:** 1 Stowers Institute for Medical Research, Kansas City, MO 64110, USA; 2 MRC Laboratory of Molecular Biology, Francis Crick Avenue, Cambridge CB2 0QH, UK; agm@mrc-lmb.cam.ac.uk; 3 Department of Molecular and Integrative Physiology, University of Kansas Medical Center, Kansas City, KS 66160, USA

**Keywords:** functional amyloids, CPEB, Orb2, huntingtin, Huntington’s disease, polyglutamine, Cryo-EM

## Abstract

Huntington’s disease is a progressive, autosomal dominant, neurodegenerative disorder caused by an expanded CAG repeat in the huntingtin gene. As a result, the translated protein, huntingtin, contains an abnormally long polyglutamine stretch that makes it prone to misfold and aggregating. Aggregation of huntingtin is believed to be the cause of Huntington’s disease. However, understanding on how, and why, huntingtin aggregates are deleterious has been hampered by lack of enough relevant structural data. In this review, we discuss our recent findings on a glutamine-based functional amyloid isolated from *Drosophila* brain and how this information provides plausible structural insight on the structure of huntingtin deposits in the brain.

Polyglutamine (PolyQ)-related diseases are dominant, late-onset genetic disorders manifested by progressive neurodegeneration. A common feature of this group of diseases, which include Huntington’s disease (HD), dentatorubral-pallidoluysian atrophy, spinobulbar muscular atrophy, and six types of spinocerebellar ataxias, is the abnormal expansion of a CAG codon repeat, coding for a 10 to 35 long glutamine tract in the wild-type protein [1]. First documented by George Huntington in 1872, HD is one of the most common inherited neurodegenerative diseases, causing cognitive disruptions and chorea with no effective cure [2]. The connection between HD and the expansion of the glutamine tract in the huntingtin (*HTT*) gene, which codes for the multidomain and multifunctional *HTT* protein [3,4], was identified in the early 1990s [5]. In HD, intraneuronal deposits of *HTT* fragments that map onto the exon 1 (*HTTex1*) are found in cerebellum, striatum, and cortex [6]. This led to the use of *HTTex1* to determine the consequences of *HTT* deposits in mice and neuronal cell lines [7,8,9,10]. HD toxicity is believed to stem from a gain-of-toxic-function of *HTT* aggregates [11], along with a loss of function through sequestration of *HTT* and other proteins into the aggregates [12,13]. However, there is an ongoing debate about the nature of the harmful proteinaceous species in the brain; prefibrillar oligomeric, generally α-helical, assemblies [14], or fibrillar amyloid assemblies [15].

In vitro, the polyQ tract encoded by *HTTex1* drives the self-assembly to an amyloid fold [16,17]. The assembly kinetics, however, also depends on the polyQ-flanking regions [18,19,20,21,22,23]. The in vitro-assembled *HTT* amyloid is proposed to adopt an antiparallel β-sheet arrangement [24,25,26,27,28,29,30,31,32,33,34]. Yet, despite intense efforts, the atomic resolution 3D architecture of aggregated *HTT*, even assembled in the test tube, remains elusive. A recent study employed cryo-ET methods to analyze the architecture of *HTTex1* amyloid-like filaments in the cellular context [35]. However, there is no atomic-level structural information of pathological polyQ aggregates from patients’ brain. Unexpectedly, the functional amyloid formed by *Drosophila* Orb2 protein, a member of the cytoplasmic polyadenylation element binding proteins, provides plausible structural insight on the endogenous polyQ-based amyloids. The aggregated state of Orb2 plays a causal role in memory stabilization [36,37,38,39,40,41]. Using cryo-EM, the structure of the biochemically active Orb2 aggregates extracted from adult *Drosophila* head have been recently solved [42]. The structure revealed that Orb2 aggregates are left-handed C3 helical amyloid filaments, defined by three molecules per layer that form, on average, 750 Å continuous in-register parallel β-sheets (Figure 1A). The filament structure is stabilized by a Q-based amyloid core (Figure 1B), while the rest of the protein, comprising the RNA-recognition motifs and protein interaction domain, extends from the Q-based amyloid core [42].

In spite of differences in β-sheets arrangement, β-parallel (in vivo-assembled Orb2) versus β-antiparallel (in vitro-assembled *HTTex1*), both proteins employ a similar arrangement of individual molecules. Of the 704 amino acids of Orb2, 31 amino acids (176-206) form an antiparallel hairpin-like structure with a hydrophilic core stabilized by 7 inter-digitated Q coming from opposing stands connected by a turn—four from one β1 strand (Q179, Q181, Q183, and Q185) and three from the opposing β2 strand (Q200, Q202, and Q204), separated by 14 residues (Q-x-Q-x-Q-x-Q-x14-Q-x-Q-x-Q motif). The interior of the Orb2 amyloid is formed by tightly packed and hydrogen-bonded Q sidechains, whereas its exterior residues contribute to the protofilament interfaces [42] (Figure 1B). Despite differences in Q-length between Orb2 and *HTTex1*, a recent study inferred a similar interdigitated antiparallel hairpin structure as the stereochemically favorable arrangement of in vitro-assembled *HTTex1* [43]. The tight interdigitation of glutamine sidechains in the packing of antiparallel β-sheets in HTTQex1 requires these sidechains to adopt two different rotamers for different strands [43], indicating structural heterogeneity at single-residue level. Specifically, the Qs of the two β-strands in the HTTQex1 β-hairpin differ in their side chain dihedral angles; however, within each strand, all Q residues are the same rotamers with the same backbone and sidechain geometry [28,43]. In this antiparallel arrangement, inter-strand hydrogen bonds are only formed between β-strands with different sidechain rotamers, but not between β-strands with the same rotamer [28,43]. On the other hand, the packing of parallel β-sheets in the Orb2 core can be achieved with the same rotamer for all interdigitating glutamines [42]. In addition, the Orb2 parallel β-structure could be stabilized by specific features of its interdigitated cross-β packing: a slight tilt of the glutamine sidechains toward the N-termini of the β-strands and the positioning of strands in one β-sheet opposite the inter-strand spaces in the other β-sheet and vice versa. These arrangements allow the formation of additional, stabilizing hydrogen bonds between the glutamine sidechains groups in one β-sheet and the carbonyl oxygen atom of main chain peptides of the opposite β-sheet, with little or no effect on peptide group conformation, as well as a tighter packing of the interdigitated glutamine sidechains from both β-sheets [42].

The Orb2 and *HTTex1* antiparallel hairpin arrangement differs in the overall amyloid architecture. The hairpin-like fold of the individual Orb2 chains is formed by the strands from two opposing parallel β-sheets [42] (Figure 2A). In contrast, the in vitro-assembled *HTT* model suggests the HTTQex1 β-hairpin is made of two hydrogen-bonded antiparallel strands in the same β-sheet [43] (Figure 2B). However, in vivo, the structure and/or arrangement of *HTTex1* β-hairpin could be dictated by context-specific factors [30], which could lead to structurally different conformations to that assembled in vitro. Indeed, activity and structure of in vitro-assembled Orb2 amyloid is distinct from Orb2 amyloid isolated from adult brain [42,44]. 

Is it possible that the endogenous, aggregated *HTT* structure is distinct from what has been inferred from in vitro studies? If so, could it be similar to Orb2 structure? Possible Orb2 and *HTT* structural similarity in the native context, besides a similar monomeric fold (i.e., hairpin) in the amyloid state, is underscored by the observation that exogenously expressed Orb2 co-aggregates with *HTTex1* in *Drosophila* motor neurons [36]. This observation could be explained by different scenarios: First, the co-localization could result from the incorporation of *HTTex1* monomers into the Orb2 filament arrangement forming a heteroaggregate, or vice versa. Second, *HTTex1* and Orb2 adopt a different structure, and the co-localization arises from a lateral surface association of *HTTex1* and Orb2 filaments. Third, endogenous *HTTex1* adopts a structure similar to Orb2. Here, the interdigitated cross-β structure observed in head-extracted Orb2 filaments could be readily extended on both sides of a parallel β-sheet made of only glutamine residues (Figure 2C). Such an arrangement would allow the formation of more stable, multilayered cross-β structures from sufficiently long polyQ sequences based on hairpins with similar β-strand lengths as minimal repeat units (Figure 2D). Indeed, contrary to earlier reports [24,45,46], ssNMR data consistently report a length-independent common structure of the polyQ amyloid [26,47]. This observation may reflect the existence of a unique polyQ structure in all polyQ diseases, where protein context (such as flanking regions), or cell-type-specific context (such as monomer availability), could determine the Qn threshold [48] and supramolecular filament polymorphism [47,49]. In the future, the elucidation of structure of *HTT* and other polyQ aggregates from diseased brains would either refute or support this thesis. 

## Figures and Tables

**Figure 1:**
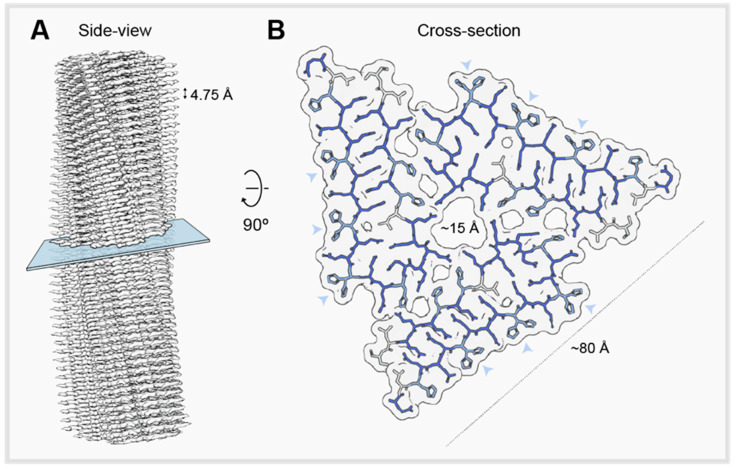
General view of the Orb2 amyloid structure. Side view of the reconstructed Orb2 amyloid core showing the ~4.75 Å separation between β-strands, typical of the amyloid fold (**A**), and cross-sectional view of one molecular layer of the calculated atomic model (**B**) [42]. Glutamines are colored in dark blue, while histidines are colored in light blue. In some histidine residues, a major and minor occupancy, alternative sidechain conformations are shown (arrow heads). Leucine and serine residues are represented in gray.

**Figure 2:**
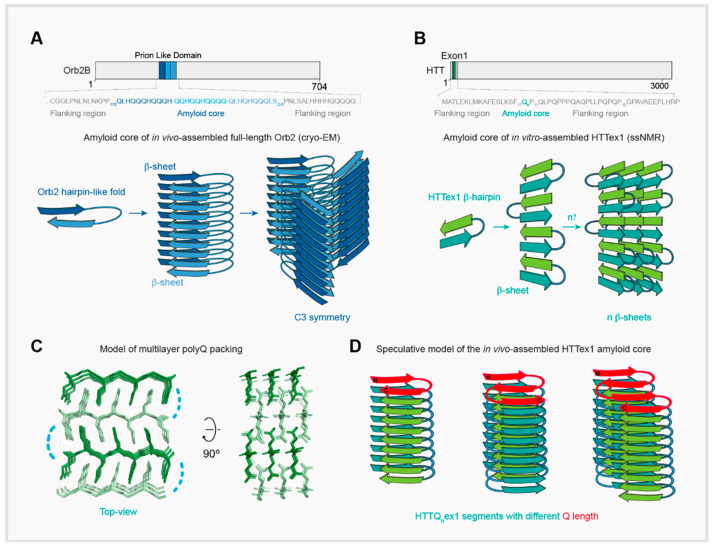
Molecular architecture of Orb2 and Huntingtin amyloids. (**A**) Schematic of the antiparallel hairpin-like fold adopted by head-extracted Orb2 filaments, derived from the cryo-EM structure. Three Orb2 molecules per molecular layer form continuous in-register parallel β-sheets. Different tone of blue represents the different amino acid composition for each β-strand of the hairpin. Amyloid forming sequence is indicated in the top. (**B**) Schematic of the antiparallel β-hairpin adopted by in vitro-assembled *HTTex1* amyloid, derived from ssNMR data. One single *HTTex1* molecule contributes to two molecular layers to form antiparallel β-sheets. Different tone of green represents the two differently structured β-strand types of the β-hairpin. (**C**) Model of a multilayer packing of the parallel polyQ β-sheets, obtained by extending the Orb2 inter-digitated cross-β structure on both sides. Blue dashed line represents the hairpin turn. The extended glutamine side chains form a steric zipper interface to allow an ~8 Å distance between β-sheets. (**D**) Proposed in vivo *HTTex1* filament model based on the multilayer polyQ packing showed in (**C**). Stacks of hairpins or meanders of similar β-strand lengths (highlighted in red), are viewed across the filament axis.

## Abbreviations

PolyQ	Polyglutamine
HD	Huntington’s Disease
*HTT*	Huntingtin
*HTTex1*	Huntingtin Exon 1
CPEB	Cytoplasmic Polyadenylation Element Binding Protein
Orb2	The *Drosophila* protein encoded by the oo18 RNA-binding (*orb*) gene
Cryo-EM	Cryo-Electron Microscopy
Cryo-ET	Cryo-Electron Tomography
ssNMR	Solid-state Nuclear Magnetic Resonance
